# Hemostatic state augmented with platelet indices among Sudanese diabetic septic foot

**DOI:** 10.1186/s12878-018-0104-5

**Published:** 2018-05-11

**Authors:** Bashir Abdrhman Bashir, Mohamed Salih Ali

**Affiliations:** Hematology department, Port Sudan Ahlia College, Port Sudan, Sudan

**Keywords:** Diabetes, Coagulation, DSF, Platelet indices, Sudan

## Abstract

**Background:**

Diabetes mellitus is a very rampant metabolic disorder, particularly type II. It has many complications such as the septic foot. Diabetic septic foot (DSF) patients are at high risk for coagulation abnormalities as well as surgical hazards. Owing to the potential sequelae of coagulation and vascular abnormalities, this work aimed at studying the hemostatic state and platelet indices in diabetes type II patients with septic foot.

**Methods:**

A case-control study was conducted during the period from July to September 2017 at Dr. Awaad medical center, Red Sea State, Sudan. 57 diabetic patients with septic foot, aged between 17 and 78 years along with 57 non-diabetic subjects as control were enrolled. Sociodemographic data were collected using a structured questionnaire. Venipuncture blood was taken with necessary safety measures. Diabetes profile, coagulation studies as well as platelet indices were estimated. Data was analyzed using SPSS version 24.0 for windows. Ethical approval was considered and written consent from each participant was obtained.

**Results:**

The mean age of diabetic patients with septic foot and healthy controls were 48.49 ± 15.8 and 32.77 ± 14.0, respectively. The duration of the diabetes onset was 10.43 ± 9.5 years. Plasma prothrombin time (PT) value (12.61 ± 2.6 vs 13.67 ± 1.5, *P* < 0.009) was found to be significantly shorter in DSF compared to control. Plasma activated partial thromboplastin time (APTT) value was significant in diabetic septic foot (32.64 ± 5.2 vs 28.49 ± 4.13, *P* < 0.000), and thrombin time (TT) did not changed in DSF. Mean platelet volume (MPV), platelet distribution width (PDW), and platelet large cell ratio (P-LCR) values were significantly decreased in DSF compared to control (*P* < 0.013, 0.034, and 0.020, respectively). PDW values were positively correlated with PT, APTT, and D-Dimer (DD) (*r* = 0.28/*p* < 0.003, *r* = 0.29/*p* < 0.029, and *r* = 0.32/*p* < 0.016, respectively). FVIII activity (121.86 ± 174.4 vs 98.66 ± 31.83, *P* < 0.951) was insignificant with DSF, as the DD was also insignificant (*P* < 0.081).

**Conclusion:**

Diabetes mellitus is associated with prothrombotic tendency. Hypercoagulable state in DSF is indicated by shortened PT finding. PDW is a manifesting evidence that proves the presence of more reactive and aggregable platelets in DSF patients.

## Background

Globally, diabetes mellitus is rising, the prevalence is steadily increasing everywhere, mostly in the middle-income countries [1]. The prevalence in rural Sudan population is about 3.9%, which in turn lead to an increase in the rate of admission [2]. Diabetic septic foot (DSF) is a common complication of diabetes, with an estimation that every 30 s a lower limb lost somewhere as a consequence of complication of DSF [3]. Eighty percent of diabetic mortality rates is due to thrombotic events. While, 75% of these mortalities belonged to cardiovascular problems and 25% remainder due to peripheral vascular events as well as cerebrovascular complications [4]. The vascular endothelium is substantially regulated of (local) hemostatic mechanism (coagulation and fibrinogen). Endothelial dysfunction has been reported in type II diabetes mellitus. A procoagulant condition is observed in diabetic patients, which ultimately contribute to cardiovascular events. Many clotting factors such as I, VII, IX, XII, Kallikrein and von Willebrand factor (VWF) are increased in diabetes [5]. This hypercoagulability could be due to an imbalance between the endothelial surface and the blood clotting factors. In type II diabetes mellitus increased level of antihemophilic factor (VIII) was observed previously with uncertain correlation to the vascular problems [6]. Some reports demonstrated insignificancy in FVIII level between diabetes type II and normal healthy individual. Thus, high levels of FVIII are more evident in the presence of increased VWF concentration [7, 8]. Platelets are normal in both diabetic and non-diabetic individuals. Recent reports have shown significant changes in platelet parameters in diabetic patients when compared with controls, particularly the mean platelet volume (MPV) and platelet distribution width (PDW) [9]. These reports suggest that platelets with variable morphology could be associated with an increased risk of vascular complications in diabetes [9]. Hyperglycemia, in turn, may contribute to endothelial dysfunction and vascular damage. This condition exerts an increase platelet reactivity and promotes glycosylation of platelet proteins [10]. This study was designed to evaluate the alteration in coagulation parameters and platelet indices and their relation to diabetic septic foot in Sudanese patients.

## Methods

### Study design and subjects

A case- control study was carried out among patients with diabetes type II presented with diabetic septic foot (DSF) at the Dr. Awaad Medical Centre (private sector clinic), Red Sea State, Sudan. 57 patients confirmed diabetic with septic foot were recruited in the study along with 57 non-diabetic healthy individual. Diabetic patient with septic foot for a long duration at least one month was enrolled in this study. Diabetic subjects who had clinical or laboratory signs of malignancy, coagulation disorders, liver impairment, cardiovascular diseases, or receiving medication that could affect the platelets and coagulation system were excluded. Prothrombin time (PT), activated partial thromboplastin time (APTT), thrombin time (TT), factor VIII, D-Dimer (DD), fasting blood glucose (FBG), glycosylated hemoglobin (Hb A1c) and platelet indices were screened in this study.

### Socio-demographic data

A structured questionnaire was utilized to collect the data from diabetic septic foot patients [11]. The questionnaire includes age, sex, duration of the disease and drug administration prior to specimen collection.

### Study sample

Open Epi calculator for case-control study was used to determine the sampling size. The standard normal variant for the power of the study is 80% where, the control exposure was measured at 23% [12]. The minimum sample size calculated for this study was 57.

### Sampling technique

A venipuncture vacuum tube method was used to collect blood sample in the morning between 8.00–9.00 a.m. after at least 12 h fasting. 3 ml added to vacutainer bottle containing di-potassium ethylene diamine tetra acetic acid (K_2_-EDTA) according to the international committee (council) of standardization in Hematology (ICSH) recommends. 3 ml was collected in fluoride oxalate vacutainer for (FBS) measured by URIT 810 semi-automated chemistry analyzer. 2.7 ml was dispensed into vacutainer bottle containing tri-sodium citrate (0.109 M) as the third sample. An aliquot of plasma was transferred into another plastic tube and promptly performed the PT, APTT, and TT (by URIT coagulometer analyzer). The remainder was frozen in − 80%°C until assayed for factor VIII and D-Dimer levels. Platelets indices were measured immediately with semi-automated hematology counter (Sysmex KX-21). Glycosylated hemoglobin was estimated by NycoCard® method using NycoCard® READER II (SN 67498, Axis-Shield PoC AS, Oslo, Norway).

### Data statistical analysis

Data of coagulation and platelet indices were estimated stepwise by comparing mean student t-test and Chi-square test. The probability value < 0.05 was taken as the minimum level of statistical significance. The findings were presented as mean ± SD. Statistical package for the social science for windows 10 (SPSS version 24, IBN, USA) was used for the statistical analysis.

## Results

Fifty-seven DFS patients 33 (57.9%) were males and 24 (42.1%) were females, with the mean age being 48.49 ± 15.8 years. Among the 57 controls 44 (77.2%) were males and 14 (22.8%) were females with the mean age being 32.77 ± 14.0 years. The laboratory characteristics for all participants, including diabetes profile, platelet indices, markers of coagulation and fibrinolysis are presented in Table 1. The duration of the diabetes was 10.43 ± 9.7 years and positively correlated with age, sex, APTT, DD, PDW, and P-LCR (*r* = 0.39/*p* < 0.003, *r* = − 0.33/*p* < 0.014, *r* = 0.27/*p* < 0.043, *r* = 0.30/*p* < 0.024, *r* = 0.31/*p* < 0.019, and r = 0.27/*p* < 0.040, respectively).

**Table 1:** Laboratory characteristics of control and diabetic septic foot

	Controls (mean ± SD)	DSF (mean ± SD)	*P*. value
Demographics			
Participants	57	57	–
Age (years)	32.77 ± 14.0	48.49 ± 15.8	0.000
Sex (Male/Female)	44/13	33/24	0.028
Duration of diabetes (years)	–	10.43 ± 9.7	–
Diabetes profile			
FBS	94.19 ± 14.2	209.01 ± 96.0	0.000
HbA1c	3.77 ± 0.57	8.36 ± 3.84	0.000
Platelet parameters			
PLT × 10^3^/μl	218.24 ± 67.6	270.78 ± 94.0	0.001
MPV fl	10.44 ± 0.99	9.95 ± 1.06	0.013
PDW fl	13.99 ± 2.34	13.07 ± 2.23	0.034
PCT %	0.227 ± 0.07	0.266 ± 0.09	0.011
P-LCR %	28.83 ± 7.30	25.61 ± 7.24	0.020
Common Coagulation tests			
PT second	13.67 ± 1.59	12.61 ± 2.57	0.009
APTT second	28.49 ± 4.13	32.64 ± 5.20	0.000
TT second	16.26 ± 2.43	16.14 ± 2.31	0.801
Special Coagulation Studies			
DD ng/ml	150.61 ± 52.98	130.81 ± 81.54	0.081
FVIII %	98.66 ± 31.83	121.86 ± 174.8	0.951

In the present study, FBG and HbA1c values were found to be significantly higher in DSF patients when compared to control (*P* < 0.000) and positively correlated with APTT (*r* = 0.37, *p* < 0.004) and negatively correlated with PT, TT, FVIII, DD, PLT, MPV, PDW, PCT and P-LCR (*r* = − 0.80/*p* < 0.386, *r* = − 0.11/*p* < 0.909, *r* = 0.17/*p* < 0.119, *r* = 13/*p* < 0.338, *r* = 0.22/*p* < 0.051, *r* = 0.18/*p* < 0.052, *r* = 0.96/*p* < 0.377, r = 0.18/*p* < 0.174, *r* = − 0.92/*p* < 0.299, respectively).

### Platelet indices

Platelet parameters in diabetic patients with septic foot in comparison to the control were found to be significantly different in PLT, MPV, PDW, PCT, and P-LCR (*P* < 0.001, 0.013, 0.034, 0.011, and 0.020, respectively). Furthermore, the MPV, PDW, and P-LCR were lower in DSF than the control. While PLT and PCT were higher in DSF as compared with the controls (Table 1).

PDW value has been recognized as an evidence of platelet activity and was significantly correlated with PT, APTT, DD, and PLT (*r* = 0.28/*p* < 0.003, *r* = 0.29/ *p* < 0.029, *r* = 0.32/*p* < 0.016, and *r* = − 0.24/*p* < 0.010, respectively). Platelet count also correlated positively with MPV, PDW, PCT, and P-LCR (*r* = − 0.29/*p* < 0.002, r = − 0.24/p < 0.010, *r* = 0.95/*p* < 0.000, *r* = − 0.30/*p* < 0.001) and negatively with PT, APTT, TT, VIII, and DD (*r* = − 0.07/*p* < 0.442, *r* = − 0.12/*p* < 0.194, *r* = − 0.10/*p* < 0.302, *r* = − 0.144/*p* < 0.284, and *r* = − 0.06/*p* < 0.638, respectively). PLT and PCT values were increased in DSF patients as compared with the non-diabetic subjects.

### Common coagulation tests

In this study, the plasma prothrombin time and activated partial thromboplastin time levels were significant in diabetic septic foot compared to the control (*P* < 0.009 and 0.000). Whereas the thrombin time coagulation test was insignificant (0.801). 50.9% of the DSF patients had shortened PT (< 12.0 s) indicating that there is a significant effect of the disease on PT results. PT value was correlated positively with APTT, PDW, P-LCR (*r* = 0.28, *P* < 0.003), and MPV (*r* = 0.20, *P* < 0.038). The statistical details of the coagulation tests are illustrated in Table 2.

**Table 2:** Findings of coagulation studies

Character/Parameters	PT (*n* = 57)	APTT (*n* = 57)	TT (*n* = 57)	FVIII (*n* = 57)	DD (*n* = 57)
Short (low)	29 (50.9%)	4 (7.0%)	–	21(36.8%)	–
Normal	25 (43.9%)	44 (77.2%)	55(96.5%)	26(45.6%)	51(89.5%)
Prolong (high)	3 (5.3%)	9 (15.8%)	2 (3.5%)	10(17.5%)	6 (10.5%)
Median	11.9	31.8	15.8	79.5	115
Range of test	10.1–25.4	24.1–44.3	13.0–25.0	14–1127	40–450
Range of control	10.0–16.5	20.1–38.9	11.0–22.2	38–456	36–200
Normal range	12–16	26–39	< 22	50–150	< 200

### Special coagulation studies

However, the concentration of DD showed decreased levels for DSF patients in comparison with the control (130.81 ± 81.54 vs 150.61 ± 52.98). In contrast, factor VIII concentration demonstrated increased levels in DSF (121.86 ± 174.8 vs 98.66 ± 31.83), although it was insignificant (*P* < 0.951) as depicted in Table 1.

## Discussion

Diabetes impairs the coagulation homeostasis which cause vascular thrombotic events [13]. In this study, we focused on the alteration of coagulation and platelet parameters that associated with the diabetic patients with septic foot. Few reports are available regarding the coagulation studies and platelet indices in diabetes, particularly in our area. The diabetic complication and its severity are depending on the duration of the disease onset [14], this observation was highlighted in our study.

With regard to platelet indices which represent as new insight in diagnosis and prediction of the platelet activity, the MPV, PDW, PCT, and P-LCR values were observed statistically significant in this study. The MPV denotes the platelet average size and activity [15]. Large platelets are younger and more potent to secrete more thromboxane A_2_ and serotonin than the small platelet [16]. The MPV and PDW were found significant in association with septic foot (microvascular injury), this may support the clue that the presence of more reactive and aggregable platelet is a factor involved in the progression of diabetes complications. These findings are similar to Alhadas and Santos et al. [15]. In fact, many authors have studied the MPV changes associated with diabetes. Among these reports, the MPV findings were controversial [16, 17]. High blood glucose values were also considered a factor that involved in augments in platelet reactivity, because it has direct effects on platelet and promotes platelet protein glycosylation [9]. However, the high MPV in diabetes is a finding that could predict thrombotic events [10]. With respect to PDW, there is an evidence in the literature that PDW is a potential marker in diabetes. Platelet activation was noted significantly, and correlated positively with PT, and DD. These results were agreed with a previous study [18] reported that PDW gives an account in diabetes on the platelet activity. The activated platelet had increased the number and size of the pseudopodia and this may possibly lead to alteration in the PDW, as expressed by Bashir et al. [15]. Moreover, many significant differences were found in PDW value in diabetic patients with septic foot when compared to other diabetic complications, a finding that is corroborated with Alhadas et al. [19] and Jindal et al. [9] and contrarily with Chen et al. who reported that there was no relation between PDW and diabetes [20]. However, the discordant findings in this study may probably be accounted to the fact that most of the diabetic had been on treatment for varying durations, particularly the antiplatelet drugs such as clopidogrel rather than the aspirin which is used constitutively to the patients, although we have excluded the patients that administrated antiplatelet medications. This could be one of the possible limitation of the study. As well, some factors may have influenced the MPV and PDW such as the platelet proliferation and turnover, platelet survival time, and poor glycemic control in type II diabetes mellitus [21].

The short result of prothrombin time and activated partial thromboplastin time predisposes the patients to high risk of thrombosis [22]. So, the risk may be increased folds on the diabetic septic foot [12]. Many researches have been demonstrated the abnormalities in the coagulation occur in association with the spectrum of diabetes mellitus, resulting a thrombophilic event [23]. A significant shortened PT results were detected in the present study in comparison with control. These findings are agreed with Erem C, et al., [5] in a study conducted in Turkey to investigate the markers of coagulation and fibrinolysis in type II diabetic patients and their results determined that the levels of fibrinogen, antithrombin, plasminogen activator inhibitor-1 (PAI-1), von Willebrand factor activity and PT were found to be significantly shorter in the type II diabetic patients in compared with the control subjects. Also, Madan et al. [24] who demonstrated that PT was short in diabetic individuals. The current study also revealed a significant difference in the activated partial thromboplastin time in diabetic septic foot patients, although most value was in the normal limits. Our results are consistent with many previous works [23, 25, 26]. The thrombin clotting time did not change in our diabetic septic foot patients, a finding that is considerably similar to Erem C, et al. [5].

FVIII has been reported to be increased in patient with type II diabetes. This augmentation may be relevant to the presence of endothelial dysfunction or inflammatory condition [27]. 95% of FVIII circulates linked with von Willebrand factor (VWF). Only 5% of FVIII circulates freely. FVIII is increased in most inflammatory states. Moreover, it is believed that this inevitably due to VWF increases, since the FVIII formation is not influenced by inflammation [28]. Elevation of FVIII levels is considered an autonomous risk factor for thromboembolism [29]. In this report, factor VIII activity showed no significant difference between diabetic septic foot patients and controls. Various studies expressed FVIII normal, increased, and decreased [13, 24, 30]. Uncontrolled diabetes acts on the fibrinolysis by stimulating the production of PAI-1. This state enhances the permanence of the clot and consequently the thrombi developed [31]. Many researches in diabetic patients revealed increased levels of DD. However, when hypercoagulability state is present there is hyperfibrinolysis consequent. Accordingly, hypercoagulability and hypofibrinolysis states are occurring in diabetic patients and the DD become underestimated [32]. Furthermore, the more rigid fibrin that result from glycated fibrinogen in a hyperglycemic patient may again contribute to decreased value, as this fibrin is difficult to be degraded [30]. DD levels in the present study are insignificant and most of values are within the normal limits. Based on the present findings, and various previous studies, the hyperglycemia has been taken to be the causative agent of abnormalities of coagulation along with vascular complication. Future studies should be expanded to evaluate platelet function, markers of endothelial injury and hypercoagulability markers.

## Conclusion

It is important to know that diabetes is associated with prothrombotic state. Hypercoagulability was indicated in DSF by shortened PT. PDW is a manifesting evidence that proves the presence of more reactive and aggregable platelets in DSF patients.

## Abbreviations

APTT: Activated partial thromboplastin time
DD: D-dimer
DSF: Diabetic septic food
FFBG: Fasting blood glucose
HbA1c: Glycosylated hemoglobin
MPV: Mean platelet volume
PAI-1: Plasminogen activator inhibitor-1
PCT: Plateletcrit
PDW: Platelet distribution width
P-LCR: Platelet large cell ration
PLT: Platelet count
PT: Prothrombin time
SPSS: Statistical package for social science.
TT: Thrombin time
VWF: Von willebrand factor
